# Lead Poison

**Published:** 1849-11

**Authors:** 


LEAD POISON.

We have reason to fear that the lead pipe is coming into more extensive use in the West than is consonant with health. Its cheapness when compared with the copper and the malleable iron pipe, the ease with which it can be bent to meet any exigency, and the ignorance of its deleterious qualities that prevails are making the lead to be quite an article of western commerce, and we think it important that western and southern physicians should be put upon their guard. The profession is familiar with the researches of Sir George Baker, Mr. Burton, John Hunter and M. Tanquerel, but their investigations were confined in a great measure to the results of the action of lead introduced into the system by manufacturing white lead, the use of it by painters, and that employed in the manufacture of cider, wines, &c. Those who may consult Tanquerel's valuable pages, will be astonished at the great number of cases of lead poison that came under his observation. But it is not our object to refer particularly to this branch of the subject. The phenomena of lead poison may be found very fully detailed in works of practice, and are familiar to all well read physicians.

The poisonous properties of *water* conveyed through leaden pipes are not so generally known, and we feel it incumbent on us to call the attention of our medical brethren to the subject. The lead pipe should not be used for the conveyance of water for man or beast, and as the profession is often consulted on subjects of this kind, we proceed to lay before it some facts which have been substantiated by an extended investigation in Massachusetts.

In the American Journal of Medical Sciences for the October quarter, we find a valuable case of lead poisoning reported by Dr. Dalton, of Boston, and he mentions, incidentally, symptoms of less gravity in other persons, than those presented in his case. It is clearly shown, that the child, whose case Dr. Dalton details, imbibed the lead poison by the use of water conveyed in lead pipes.

Dr. Dana, of Lowell, Massachusetts, in a pamphlet quoted by Dr. Dalton, has conclusively established the fact that the lead pipes which convey water in that city are often so extensively corroded, that they need the services of the plumber. Dr. Dalton says: "I have in my possession a piece of lead pipe from one of these wells, [at Lowell] the surface of which is irregularly corroded, as if worm-eaten, over its whole extent, and at several points complete perforation has taken place. This fact, therefore, that lead pipes are corroded and perforated by the water of some wells, is a sufficient refutation of the theory of its protection by saline matter, however respectable may be the authority by which this theory is advanced. In one of Sir George Baker's earliest communications to the London College of Physicians on this subject, there is a letter from a Dr. Wall, who gives an account of a certain family in his neighborhood, of whom the mother was paralytic, and the children all subject to disorders of the bowels. Several of the family died, and the house was sold. The next occupant found the 'leaden pump' belonging to the premises in so leaky a condition, from corrosion of the lead, that it was necessary to repair it, and the pump maker who was employed said that he had several times been obliged to do the same thing, on account of the same difficulty, during the stay of the original occupants." The facts on this subject are too numerous to permit any doubt as to the deleterious effects of water conveyed in lead pipes, and it should not be forgotten that cows, horses and sheep are as susceptible of the poisonous influence as man. Those who are familiar with the history of the "lead hills" of Scotland, are in possession of many valuable facts as to the effect of lead upon the lower order of animals.

Professor Horsford, of Cambridge University, U. S., has contributed a valuable paper on "the action of water on leaden service pipes." It is valuable on account of the details given for the detection of the presence of small quantities of lead in water, but we cannot now spare the space for these details. We return to Dr. Dalton's valuable paper. He says: "Dr. Dana ascertained that the natural waters of Lowell all contain saline ingredients, the most abundant of which are sulphate of iron, nitrate of lime, and chloride of sodium; and that the waters of the dis trict called Chapel Hill, [in which cases of lead poisoning were numerous and severe] abounded chiefly in sulphate of iron. Dr. Dana concluded that, if natural waters contained only one salt, the exact proportion and protective action of which could be easily ascertained, the whole matter would be very simple;—but in point of fact, there are several salts existing together in solution in well water, very often in company with vegetable acids; and that these act on each other, and modify each other's action on the lead."

"The dangerous results liable to ensue from this mutual action are principally three-fold.

"I. The production of chloride of lead, which is soluble in water.

"II. The production of nitric acid, which readily dissolves lead, &c.

"III. The production of carbonate of lead, which, though insoluble, is liable to be introduced into the system in a state of suspension, and is, therefore, nearly or quite as dangerous as if soluble."

These are the sources of danger, and should be attended to by medical men. And Liebig's suggestion should not be forgotten—that persons exposed to the dangerous action of lead, may find a protection in sweetened water, acidulated with sulphuric acid.

There can be no apology for the use of the lead pipe. The great improvements made in the manufacture of cast iron pipe render the latter a perfectly eligible article for the uses to which the lead pipe has been appropriated. In the large establishment of P. R. Gray & Son, in this city, cast iron pipes are made that may follow as crooked and winding a path over a house as lead can be made to do, and they make a more ornamental work. Whatever the cast iron pipe may impart to water conveyed through it, we know to be salutary, and the difference between the lead and cast iron pipe for conveying water, is the difference between a poison and a tonic, between death and life. B.

